# Detection of Static and Dynamic Stereopsis after Femtosecond Laser Small Incision Lenticule Extraction for High Myopia

**DOI:** 10.1155/2021/6667263

**Published:** 2021-06-11

**Authors:** Aiqun Xiang, Chu Hang, Xiaoying Wu, Yewei Yin, Yanyan Fu, Ying Lu, Kaixuan Du, Tu Hu, Li Yan, Dan Wen

**Affiliations:** ^1^Eye Center of Xiangya Hospital, Central South University, Changsha, Hunan, China; ^2^Hunan Key Laboratory of Ophthalmology, Changsha, Hunan, China; ^3^National Engineering Research Center for Healthcare Devices, Guangzhou, Guangdong, China

## Abstract

**Purpose:**

The purpose of this study is to test binocular visual function after femtosecond laser small incision lenticule extraction (SMILE) for high myopia. The traditional Titmus stereotest and dynamic stereotest based on the visual perception biological model were used for comparative analysis.

**Methods:**

A total of 43 patients were enrolled in this prospective study. At Week 1, Month 1, and Month 3 after surgery, the Titmus stereotest and dynamic stereotest generated by MATLAB were conducted. Dynamic stereopsis consists of randomly flickering Gabor spots and is divided into two models of high energy and low energy according to flicker frequency.

**Results:**

The preoperative manifest refraction spherical equivalent was −7.21 ± 0.70 *D*. The preoperative anisometropia was 0.52 ± 0.54D. The quartiles of static stereoacuity in preoperation and 3 follow-ups were as follows: 50.00 (25.00, 100.00) in preoperation, 63.00 (40.00, 63.00) at Week 1, 40.00 (32.00, 63.00) at Month 1, and 40.00 (25.00, 50.00) at Month 3. Static stereopsis improved at Month 1 and Month 3 compared with preoperation and Week 1 (*P* < 0.05). There were statistically significant differences in high energy dynamic stereopsis at Week 1 and Month 1 compared to preoperation (*P* < 0.05). In addition, significant differences in low energy dynamic stereopsis were detected between Month 1 and preoperation and also at Month 3 compared to Month 1 (*P* < 0.05).

**Conclusion:**

Most high myopia patients have a dynamic stereopsis deficiency before refractive correction. SMILE surgery can improve both static and dynamic stereopsis early in the postoperation period. However, in the long term, there is no significant difference or even a decrease in dynamic stereopsis.

## 1. Introduction

Stereopsis (also known as three-dimensional vision or depth perception) refers to the perceptual ability to accurately judge the three-dimensional spatial positions of objects [1, 2]. It is an advanced binocular visual function which is not only based on binocular stimulus and fusion function, but also on visual perception by brain neural networks [3, 4]. Stereopsis is an important component of binocular visual function which can guide us in undertaking a wide range of intricate tasks. In clinical studies, Titmus stereotest and random point examination are widely used to examine stereoscopic function, but these methods cannot be used to measure dynamic or more advanced stereoscopic vision. Myopia is one of the most common eye diseases in humans [5]. The number of patients with high myopia is increasing, and many high myopia patients have defects in their binocular vision [6, 7]. At present, surgery has become a better choice for high myopia correction. Femtosecond laser small incision lenticule extraction (SMILE) is a safe and effective refractive surgery for myopia [8, 9], and whether the visual acuity restored after surgery improves stereopsis, especially dynamic stereopsis which is closely related to daily life, is exactly what we need to observe.

With the development of brain visual science, we have a further understanding of the visual processing mechanism. Meanwhile, the visual perception examination and treatment system platform based on neurophysiological theories enriches detection methods for binocular function impaired and provides a basis for accurate diagnosis and treatment. In this study, we adopted a dynamic stereoscopic biological model based on the MATLAB algorithm designed by the National Engineering Research Center for Healthcare Devices, which can help us to examine stereopsis in multiple dimensions [10, 11].

## 2. Materials and Methods

### 2.1. Patients

A total of 43 high myopia patients (12 men and 31 women) with a mean age of 25.48 ± 5.18 years (18–38 years) who underwent SMILE between May 2019 and March 2020 at the Laser Center of the Department of Ophthalmology, Xiangya Hospital of Central South University, were recruited in this study. The inclusion criteria were patients with myopia of over −6.00 diopters (D), age of 18 years or more, corrected distance visual acuity (CDVA) of 20/20 (Snellen) or better, and stable refraction for 2 years; patients with other eye diseases and significant coexisting ocular abnormalities such as cataracts or previous surgical history were excluded. All patients completed written informed consent before inclusion in the study. All study protocols were approved by the Medical Ethics Committee of Xiangya Hospital of Central South University and carried out in adherence to the Declaration of Helsinki regarding ethical principles for research involving human subjects.

### 2.2. Surgical Procedure

A VisuMax femtosecond laser system (Carl Zeiss Meditec, Jena, Germany) was used to perform SMILE. The following femtosecond laser parameters were used: 120 *μ*m cap thickness, 7.0∼7.6 mm diameter of the cap, and 6.0∼6.8 mm diameter of the posterior lenticule surface. A 2 mm long corneal incision was made at the 10 o'clock position. A microseparator was used to separate and remove the lenticule. Standard SMILE procedures were performed by the same surgeon (DW) for all patients. Medication was received as follows: 0.3% tobramycin eye drops, tobramycin dexamethasone eye drops, pranoprofen eye drops, 0.1% fluorometholone solution, and nonpreservative artificial tears.

### 2.3. Measurement of Visual Indicators

Uncorrected distance visual acuity (UDVA) was routinely measured at every visit, and time-course changes in CDVA after surgery were obtained from the clinical records. CDVA [12] was determined using a standard visual acuity chart and then converted into a logarithm of the minimal angle resolution (logMAR) visual acuity for statistical analyses. The measurements were conducted four times: preoperation, Week 1 after surgery, Month 1 after surgery, and Month 3 after surgery.

### 2.4. Measurement of Stereoacuity

#### 2.4.1. Titmus Stereoacuity Test

All subjects wore polarized glasses with refractive correction. The image disparity of the Titmus stereotest varied from 3,000 (the wingtips of the stereo fly) to 20 arcsec (tenth test circle). Subjects were measured at a distance of 40 cm. The Titmus fly was initially presented in order to ensure that the patients had stereopsis function. Passers were followed in the next check, recognizing circles 1–10 on the basis of the graded arcsec indicated on the test items, and the minimum stereoacuity was recorded.

#### 2.4.2. Dynamic Stereoacuity Test

**Figure 1:**
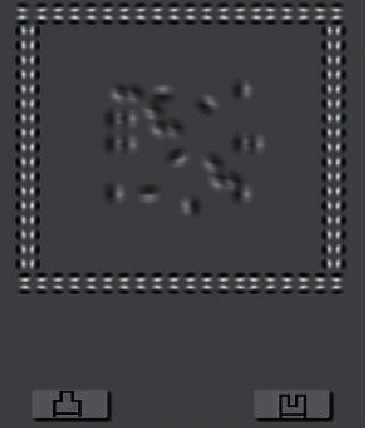
RDK dynamic stereopsis. Patients wearing polarized glasses were asked to recognize that the outlines of letter “N” were elevated from or flat on the screen taking the square as a reference. There were two modes: high energy dynamic stereopsis and low energy dynamic stereopsis. The results are qualitatively observed with yes or no, which were recorded as “1” and “0”.

After refractive correction with spectacles, all subjects wore polarized glasses to observe the stimulus on a screen with a grey background (44 cd/m^2^). The stimulus was a square containing 16 Gobar spots generated by a random-dot kinematogram (RDK) algorithm. These Gobar spots formed two outlines of the letter “N” according to the motion definition structure and were displayed to the two eyes through the polarized glasses. The two letters had binocular parallax and could be fused to form stereoacuity. The energy level was the main variable, decided by the flicker frequency. The high energy dynamic stereoacuity was stimulated by the RDK with the monitor frame rate of 10 Hz and the low energy with the monitor frame rate of 5 Hz. Subjects were asked to use the square as a reference to recognize whether the outlines of the letter “N” were elevated or flat relative to the screen. The results were qualitatively observed with yes or no responses, which were recorded as “1” and “0” (Figure 1).

#### 2.4.3. Statistical Analysis

Statistical analysis was performed using SPSS Statistics for Windows (Ver. 22.0.; IBM Corp.; Armonk, NY). Normally distributed data was presented with mean ± standard deviation. Abnormally distributed data was presented as median (P25, P75). Visual acuity and spherical equivalent were examined by the paired sample *t*-test. A comparison of stereopsis by the Titmus test was made using a Wilcoxon signed-rank test, and a *P* value of <0.05 was considered a statistically significant difference. A comparison of RDK dynamic stereopsis was made using a *χ*2 test, and a *P* value of <0.05 was considered a statistically significant difference.

## 3. Results

### 3.1. Study Subjects

A total of 43 patients aged 18 to 38 were enrolled in this study. The mean age was 25.48 ± 5.18 years. Each of the 43 subjects underwent SMILE in both eyes. The preoperation CDVA at 5 m of all patients was 20/20 or better. Compared with preoperation, there were statistically significant differences in UDVA and CDVA at Week 1, Month 1, and Month 3 after the operation (*P* < 0.05). The mean preoperation manifest refraction spherical equivalent (SE) was −7.21 ± 0.70 *D*. The SE of the right eye was −7.22 ± 0.64 *D*, and the left eye was −7.20 ± 0.76 *D*. The mean anisometropia in preoperation was 0.52 ± 0.54D, which reduced in the three follow-ups (*P* < 0.05). The changes of the visual acuity and refractive error are summarized in Table 1.

### 3.2. Comparison of Stereoacuity before and after Operation

**Figure 2:**
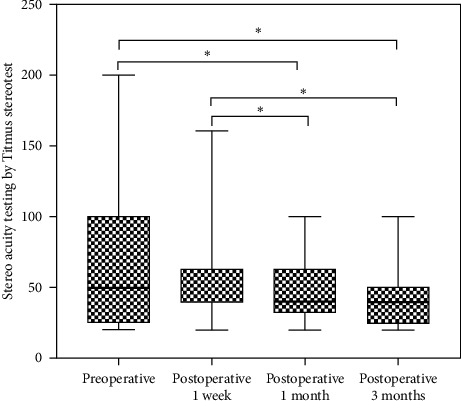
Box plot showing the level of stereopsis of each time testing by Titmus stereotest. Error bars showed the maximum or minimum. ^*∗*^Statistically significant difference.

**Figure 3:**
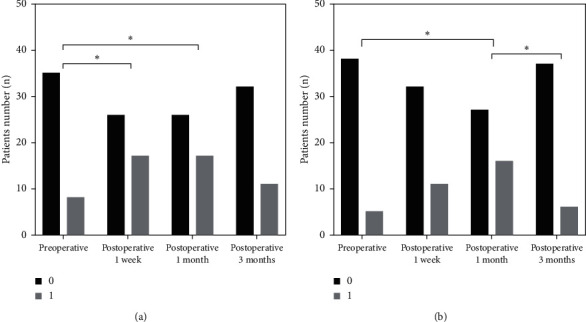
Bar graph showing the patients number who have the RDK stereopsis of each time. (a) High energy dynamic stereopsis and (b) low energy dynamic stereopsis. ^∗^Statistically significant difference.

The quartiles of static stereoacuity in preoperation and 3 follow-ups were as follows: 50.00 (25.00, 100.00) in preoperation, 63.00 (40.00, 63.00) at Week 1, 40.00 (32.00, 63.00) at Month 1, and 40.00 (25.00, 50.00) at Month 3. After surgery, static stereopsis was much higher than preoperation at Month 1 (*P*=0.022) and Month 3 (*P* < 0.001) (Figure 2), while the differences between Week 1, Month 1, and Month 3 were statistically significant, respectively (*P*=0.001, *P* < 0.001) (Figure 2). There were significant differences in high energy dynamic stereopsis at Week 1 and Month 1 after surgery compared to preoperation, respectively (*P*=0.033, *P*=0.033) (Figure 3), but there was no significant difference at Month 3 (*P* > 0.05). Low energy dynamic stereopsis was statistically significant between Month 1 after surgery and preoperation (*P*=0.006), and also at Month 3 compared to Month 1 after surgery (*P*=0.013) (Figure 3).

## 4. Discussion

Stereopsis is an advanced visual function established on the basis of visual stimulus and binocular vision. It is not possessed at birth but gradually acquired with the increase of visual experience after birth. For human stereopsis, the critical period is about 3 months of age; it then develops at a rapid rate and becomes mature at around 8–12 months of age, followed by a continued gradual improvement in stereoacuity until at least 3 years of age [13, 14]. Many patients with strabismus, amblyopia, and high myopia have stereopsis defects. There have been many studies on binocular visual function in corneal refractive correction surgery. Stereopsis is an important component of binocular visual function that has been largely overlooked in the field of refractive surgery [15]. As an ever-increasing emphasis is placed on the importance of stereopsis in our daily life, it is imperative to evaluate the effects of refractive correction surgery on stereoscopic vision. In clinical studies, most of what we examine is static stereopsis, but most of the things we observe in daily life are dynamic. Therefore, in this study, we used the Titmus stereotest as the old method of static stereotest and used a MATLAB algorithm to generate a new method of dynamic stereotest with adjustable parameters [11, 16]. The combination of the two methods was used to evaluate the stereopsis of high myopia after SMILE.

There are different research results in the previous studies on the stereoacuity changes after corneal refractive surgery. Several studies provided results that myopic patients who underwent laser in situ keratomileusis (LASIK) have increased stereoacuity, which may be due to the degree of anisometropia [17–19]. While an investigation by Siamak et al. in 2016 provided results with stereoacuity deteriorated after photorefractive keratectomy (PRK) [20], another investigation by Samrat et al. in 2020 found that stereoacuity was impaired after LASIK surgery [21]. They reported that deterioration of the stereoacuity may be mainly due to high-order aberration, corneal related complications, and the differences of two eyes [20, 21]. In our study, we found that most high myopia patients have a dynamic stereopsis deficiency before refractive correction, patients with Titmus stereotest and high energy RDK dynamic stereopsis improved after SMILE surgery compared with preoperation, and low energy RDK dynamic stereopsis improved first and then declined after surgery.

Wolfe and Held believed that stereoscopic vision is formed via the binocular process [22]. As the intermediate station of visual information, the eyes receive external information and transmit the visual information to the visual cortex. When suffering from eye diseases (strabismus, amblyopia, high myopia, etc.), the cerebral cortex suffers developmental abnormalities in perception, fusion, and stereo vision, resulting in defects in visual signal processing. Most high myopia patients live with insufficient correction for a long time, so when they look at distant objects, the image on the retina is blurred. Second, compared to wearing glasses, objects are smaller than they appear without glasses. Blurred and smaller images cause less stimulation to the retina, so the impulse entering the visual cortex is lower, and the excitability of the visual cortex to the slight differences between myopia and normal people is reduced. As a result, depth perception will become worse and a low level of stereo vision will be shown [23].

In clinical studies, we usually use the Titmus stereotest to check stereoacuity [24]. In this study, as measured by the Titmus test, we found that patients had improved static stereopsis after surgery. Studies in psychophysics and neurobehavioral physiology have shown that stereopsis is processed on the dorsal, occipital, ventral, and occipital-temporal channels, while static stereopsis is biased toward the processing of the ventral channel [25]. Random-dot kinematograms (RDKs) have been used widely in vision research as a tool for studying the mechanisms involved in human motion perception. Under such a stimulus, a dense array of random dots is displaced over both space and time, and the ability to discriminate the correct direction of coherent dot motion is measured as a function of certain stimulus parameters (e.g., spatial and temporal displacement, dot size, dot density, contrast, spatial frequency content, etc.) to elucidate the nature of the underlying processes mediating motion perception [26, 27]. In the visual perception test platform based on the MATLAB algorithm, dynamic stereopsis was designed on the theory of RDK, which is more complexed and advanced than static stereopsis, and the main processing was the dorsal channel. Our primary findings show that static stereopsis and RDK dynamic stereopsis improved in the early postoperation period. It may explain as the following reasons: after SMILE surgery, visual acuity got better and the degree of anisometropia in the eyes reduced, so the retinal imaging is clearer and the binocular parallax may become smaller which increased the stimulation of visual signals transmitted from these two pathways to the cerebral cortex, in turn enhancing the fusion function of the brain and improving stereoacuity. However, the low energy dynamic stereopsis began to decline after a long period of time. Previous studies have confirmed that stereoacuity improves with the duration of stimulus presentation [28]. This result may indicate that dynamic stereopsis is more delicate and complicated than static stereopsis; SMILE surgery only changes the imaging clarity of the retina and produces a short-term stimulus to the brain. However, the structure and function of the brain are not improved, and the improvement of dynamic stereopsis requires more time and visual experience. Whether we can make this kind of improvement permanent and stable through visual plastic training and the importance of low energy dynamic stereopsis to restore visual quality after refractive surgery are worth studying and exploring in future research.

## Figures and Tables

**Table 1:** Summary demographic of patients in the study (mean ± SD).

		Preoperative	Postoperative 1 week	Postoperative 1 month	Postoperative 3 months
UDVA (logMAR)		1.30 ± 0.27	−0.08 ± 0.09^*∗*^	−0.12 ± 0.09^*∗*^	−0.11 ± 0.09^*∗*^
CDVA (logMAR)		−0.16 ± 0.04	−0.13 ± 0.06^*∗*^	−0.17 ± 0.03	−0.16 ± 0.04
Mean SE (*D*)	OD	−7.22 ± 0.64	−0.13 ± 0.25^*∗*^	−0.11 ± 0.30^*∗*^	−0.15 ± 0.30^*∗*^
	OS	−7.20 ± 0.76	−0.02 ± 0.30^*∗*^	0.01 ± 0.32^*∗*^	−0.02 ± 0.33^*∗*^
Anisometropia (*D*)		0.52 ± 0.54	0.24 ± 0.21^*∗*^	0.20 ± 0.20^*∗*^	0.24 ± 0.28^*∗*^

UDVA = uncorrected distance visual acuity, CDVA = corrected distance visual acuity, logMAR = logarithm of the minimum angle of resolution, SE = spherical equivalent, *D* = diopters.^*∗*^ VS: preoperative, *P* < 0.05.

## Data Availability

The raw data used to support the findings of this study are available from the corresponding author upon request.
